# "Hygienenetzwerk Südostniedersachsen" – a model for hygiene network formation in Germany

**DOI:** 10.1186/1753-6561-5-S6-P37

**Published:** 2011-06-29

**Authors:** S Pfingsten-Würzburg, M Plischke, R Schubert, W Bautsch

**Affiliations:** 1 Local Health Authority, Germany; 2 BITZ Braunschweig Informatics and Technology Center, Germany; 3 City Hospital Brunswick, Braunschweig, Germany

## Introduction / objectives

Regional network formation is an integral part of any strategy to combat antimicrobial resistance development, as recommended by the German Antimicrobial Resistance Strategy (DART). But no information about the adequate organization in decentralized health systems such as Germany is given. Here, we report on the `Hygienenetzwerk SüdostniedersachsenÂ´ (HN-SON), a regional network in Lower Saxony, Germany, with special focus on organizational aspects.

## Methods

Starting in 2009, a hygiene network of local health care providers was founded in Brunswick, Germany, moderated by the local health authority and focusing around the City Hospital, a major academic teaching hospital of >1000 beds.

## Results

1. A hygiene network is an important source of information for many academic institutions, who may themselves offer important services free-of-charge in exchange. Thus, HN-SON soon cooperated with several institutions such as eHealth.Braunschweig on e. g. electronic patient admission-discharge management. 2.The majority of patient transferrals takes place within the catchment area of the major regional hospital. With HN-SON, this covers 8 health authority districts which soon cooperated in HN-SON. 3. HN-SON started as an informal association, an organization prohibitive to many activities, such as clinical studies, acceptance of donations and the cooperation with companies. We are currently founding a registered society which shall subsequently be incorporated into HN-SON.

## Conclusion

These developments will hopefully expand our possibilities (as a juristic person) to offer better regional hygiene services, such as specialized training courses or a central help desk.

## Disclosure of interest

None declared.

